# Unraveling the link between hypertriglyceridemia, dampness syndrome, and chronic diseases: A comprehensive observational study

**DOI:** 10.1097/MD.0000000000039207

**Published:** 2024-08-16

**Authors:** Hui Zhou, Weizheng Zhang, Xiangsheng Cai, Shuo Yang, Aolin Liu, Xiaowen Zhou, Jianxiong Cai, Darong Wu, Hongli Zeng

**Affiliations:** a Department of Health Assessment and Intervention, Guangzhou Cadre and Talent Health Management Center, Guangzhou 11th People’s Hospital, Guangzhou, China; b The Second Clinical Medical College, Guangzhou University of Chinese Medicine, Guangzhou, China; c State Key Laboratory of Dampness Syndrome of Chinese Medicine, The Second Affiliated Hospital of Guangzhou University of Chinese Medicine, Guangzhou, China; d Guangdong Provincial Key Laboratory of Clinical Research on Traditional Chinese Medicine Syndrome, The Second Affiliated Hospital of Guangzhou University of Chinese Medicine, Guangzhou, China.

**Keywords:** chronic noncommunicable diseases, correlation, dampness syndrome, hypertriglyceridemia, lifestyle

## Abstract

To investigate the dampness syndrome score in hypertriglyceridemia and the correlations between hypertriglyceridemia and other chronic diseases and lifestyle factors. Data were retrospectively obtained from individuals who underwent physical examinations at Guangzhou Cadres Health Management Centre from May 2022 to May 2023. *t* Test, variance analysis, and chi-square test were used to compare the score of dampness syndrome and the prevalence of hypertriglyceridemia among different subgroups. Pearson, Spearman correlation analysis, and regression analysis were used to explore the correlations between hypertriglyceridemia and dampness syndrome, chronic diseases, and lifestyle factors. The prevalence of hypertriglyceridemia was 26.70%. Clinical test index and dampness syndrome score were significant differences between hypertriglyceridemia group and normal group (*P* < .05). Subgroup analyses as a function of the degree of triglyceridemia indicated that the dampness syndrome score increased with increasing degree of triglyceridemia (*P* < .05). Correlation analysis showed that hypertriglyceridemia was correlated with dampness syndrome, overweight/obesity, hypertension, diabetes, and other chronic diseases (*P* < .05). Multivariate logistic regression analysis showed that age, sex, marriage, education level, smoking, drinking, fruit consumption, vegetable consumption, milk and dairy product consumption, dessert or snack consumption, the degree of dampness syndrome, and engagement in exercise were associated with hypertriglyceridemia (*P* < .05). Hypertriglyceridemia is associated with a variety of chronic diseases and lifestyle factors, and is closely related to dampness syndrome. The score of dampness syndrome can reflect hypertriglyceridemia to a certain extent. It provides more clinical reference for the treatment of hypertriglyceridemia combined with the analysis of dampness syndrome of traditional Chinese medicine.

## 1. Introduction

Hypertriglyceridemia is a common type of dyslipidemia and a risk factor for a variety of diseases, including atherosclerotic cardiovascular disease and acute pancreatitis.^[1,2]^ A Korean cohort study suggested that the triglyceride (TG) level is an independent predictor of cardiovascular events in young adults (20–39 years) who have not taken statins; the risk of cardiovascular events was increased by 20% with TG levels in the highest quartile.^[3]^ Previous studies have shown that residual cardiovascular risk persists even when low-density lipoprotein cholesterol (LDL-C) is reduced to the recommended target, and this residual risk may be attributed to atherosclerotic dyslipidemia, including elevated TGs.^[4]^ Previous studies have also demonstrated a positive correlation between elevated TG levels and increased risk of cardiovascular disease and mortality.^[5,6]^ This association is endorsed by the American Heart Association.^[4]^

Within traditional Chinese medicine (TCM) theory, “dampness syndrome” refers to a category of symptoms caused by dampness invading the muscles, meridians, bones and joints, paralyzing and blocking “qi” and blood flow, or internal viscera stagnation of “qi”,^[7,8]^ in which the human body is in a state of disease. The occurrence of dampness syndrome may be closely related to the geographical environment, body dysfunction, and abnormal body fluid metabolism.^[9,10]^ According to TCM theory, dyslipidemia has certain similarities with the expressions of “blood turbidity” “cream fat” and “fat man”, and such diseases are mostly attributed to dampness. Zhang et al^[11]^ analyzed 196 patients with dyslipidemia in Guizhou Province and found that damp heat and phlegm-dampness were evident in the highest proportions of these patients, suggesting that they may act as risk factors for dyslipidemia. Zhang and Lu^[12]^ found that the TG level of patients with phlegm-dampness hyperlipidemia was higher than that of other groups. Together, these findings suggest that dampness syndrome may also be a risk factor for hypertriglyceridemia (HTG). “Preventing diseases before they occur” and “three-cause approach” are the essence of Chinese medicine in preventing and treating diseases. In order to construct the treatment strategy of HTG in the field of TCM, it is crucial to explore the relationship and distribution characteristics of dampness syndrome with HTG and chronic diseases. Thus, it is worth studying whether the “dampness” characteristic of the human body can serve as an intuitive, noninvasive, low-cost, and timely indicator of abnormal blood lipids. Systematic studies on the relationship between dampness syndrome of TCM, HTG, and chronic diseases are still rare so far. This study may provide an important reference basis for the study of the relationship between the three. To this end, this study aimed to characterize dampness syndrome in the HTG population, analyze the lifestyle risk factors for HTG, and examine the correlations between HTG, dampness syndrome, and chronic diseases. The ultimate goal was to provide health guidance for the HTG population and data to support interventions and treatments for HTG.

## 2. Participants and methods

### 2.1. Subjects

Data were retrospectively obtained from public officials who underwent physical examinations and completed the TCM dampness syndrome evaluation scale at Guangzhou Cadres Health Management Centre from May 2022 to May 2023. A total of 7701 individuals with complete data were included in the analyses. The sample ranged in age from 22 to 75 years, with an average age of 44.88 ± 11.55 years. This study was approved by the Ethics Committee of Guangzhou Cadre Health Management Centre. All participants provided written informed consent.

### 2.2. Diagnostic criteria

The diagnostic criteria for HTG were as follows^[13]^: based on the fasting TG level, TGs were classified as marginal elevation (≥1.7 and <2.3 mmol/L), mild to moderate elevation (≥2.3 and <5.6 mmol/L), or severe elevation (≥5.6 mmol/L). The degree of dampness syndrome^[14]^ was evaluated according to the Diagnostic Evaluation Scale of TCM Dampness Syndrome issued by the State Key Laboratory of Dampness Syndrome of Chinese Medicine. The scale is a self-rating scale for patients with 30 items, and each item is classified into 5 grades according to the degree of zero, mild, moderate, severe, and extremely severe, which are 0, 1, 2, 3, and 4 points respectively, shown in Table S1, Supplemental Digital Content, http://links.lww.com/MD/N349. The maximum score on the scale is 120 points; the higher the score, the more serious the degree of dampness syndrome. Scores of 0–19 points are classified as “no dampness syndrome”; 20–39 points are classified as “mild dampness syndrome”; 40–59 points are classified as “moderate dampness syndrome”; 60–79 points are classified as “severe dampness syndrome”; ≥80 points are classified as “extremely severe dampness syndrome.”

### 2.3. Inclusion and exclusion criteria

The inclusion criteria were as follows: completed the TCM Dampness Syndrome Evaluation Scale and obtained a score ≥0; was able to provide written informed consent, cooperate with the completion of the questionnaire and provide a blood sample; aged ≥20 years old and ≤75 years old. The exclusion criteria were as follows: cannot cooperate with the project; patients with mental illness; pregnant or lactating women; patients with serious primary heart, liver, kidney, and other diseases of key organs.

### 2.4. Methods

Participants completed a survey, physical examination, and laboratory blood tests. All data were extracted from the physical examination system of the health management center. We randomly selected participants among those who came for medical checkups and arranged for trained investigators to assist in questionnaire completion to ensure the completeness and authenticity of data collection to minimize bias. During the data analysis of this study, there were no missing data except for insufficient response. The questionnaire included: general demographic information (including age, gender, education level, etc); lifestyle assessment (including smoking, drinking, etc); disease history; TCM Dampness Syndrome Assessment Scale and baseline questionnaire. The physical examination included: height, body mass, body mass index (BMI), heart rate, systolic blood pressure (SBP), diastolic blood pressure (DBP), and waist circumference. The laboratory test indicators included: blood lipids (total cholesterol, TG, LDL-C, high-density lipoprotein cholesterol), fasting blood glucose (FBG), liver function (aspartate aminotransferase, alanine aminotransferase, and glutamyl transpeptidases) and renal function (blood urea nitrogen, serum creatinine, and blood uric acid [BUA]), etc.

### 2.5. Statistical methods

SPSS 26.0 statistical software was used for the statistical analysis of the data. The measurement data were expressed as the mean ± standard deviation (χ ± s). *t* Tests, variance analysis, and chi-square tests were used for comparisons between groups. Pearson correlation analysis was used for normally distributed variables and Spearman correlation analysis was used for nonnormally distributed variables. Count data were compared by chi-square tests. The factors influencing HTG were analyzed by binary logistic regression analysis. *P* < .05 was considered statistically significant.

## 3. Results

### 3.1. Prevalence of HTG

Among the 7701 physical examination subjects, 2056 individuals (26.70%) were found to have HTG, including 1057 (51.41%) with marginal elevation, 887 (43.14%) with mild to moderate elevation, and 112 (5.45%) with severe elevation. The sample comprised 1673 (34.6%) males and 383 (13.4%) females with HTG; there was a statistically significant difference in the prevalence of HTG between males and females (χ^2^ = 415.26, *P* < .001).

### 3.2. Comparison of the physical examination indices between the HTG group and normal group

The differences between the 2 groups are shown in Table 1. The mean age, waist circumference, BMI, SBP, DBP, BUA, serum creatinine, blood urea nitrogen, FBG, TG, LDL-C, alanine aminotransferase, aspartate aminotransferase, glutamyl transpeptidase and dampness syndrome score in the HTG group were higher than those in the normal group. The high-density lipoprotein cholesterol level was lower in the HTG group than in the normal group (*P* < .05). There was no significant difference in the mean heart rate between the 2 groups (*P* = .514).

**Table 1:** Comparison of physical examination indices between the hypertriglyceridemia group and normal group.

Variable	Normal group (5645)	Hypertriglyceridemia group (2056)	*t*	*P* value
Age	44.07 ± 11.89	47.10 ± 10.23	−10.98	<.001
WC	79.49 ± 9.25	87.32 ± 8.01	−36.97	<.001
HR	71.40 ± 5.86	71.59 ± 5.98	−1.24	.216
BMI	23.45 ± 3.06	25.78 ± 2.93	−30.51	<.001
SBP	118.26 ± 14.76	125.56 ± 14.49	−19.26	<.001
DBP	70.29 ± 10.13	76.14 ± 10.56	−21.69	<.001
BUA	375.61 ± 95.49	445.65 ± 99.72	−28.14	<.001
SCr	75.65 ± 20.81	82.92 ± 27.53	−10.89	<.001
BUN	5.14 ± 1.30	5.22 ± 1.27	−2.44	.015
FBG	5.21 ± 0.69	5.55 ± 1.15	−12.55	<.001
TC	4.94 ± 0.90	5.37 ± 0.99	−17.39	<.001
LDL-C	2.95 ± 0.79	3.19 ± 0.89	−10.80	<.001
HDL-C	1.57 ± 0.35	1.25 ± 0.24	45.62	<.001
ALT	21.67 ± 17.32	30.77 ± 20.86	−17.69	<.001
AST	20.18 ± 13.31	22.38 ± 9.10	−8.24	<.001
γ-GT	22.56 ± 20.96	39.47 ± 35.19	−15.70	<.001
Dampness syndrome score	24.45 ± 16.22	26.39 ± 17.23	−4.56	<.001

ALT = alanine aminotransferase, AST = aspartate aminotransferase, BMI = body mass index, BUA = blood uric acid, BUN = blood urea nitrogen, DBP = diastolic blood pressure, FBG = fasting blood glucose, HDL-C = high-density lipoprotein cholesterol, HR = heart rate, LDL-C = low-density lipoprotein cholesterol, SBP = systolic blood pressure, SCr = serum creatinine, TC = total cholesterol, WC = waist circumference, γ-GT = glutamyl transpeptidases.

### 3.3. Comparison of dampness syndrome scores in people with triglyceridemia

The dampness syndrome scores of the sample as a function of the level of triglyceridemia are shown in Table 2. The dampness syndrome scores increased with increasing degree of triglyceridemia (*P* < .05). However, there was no significant increase in the severely elevated group compared with the mild-moderate elevated group.

**Table 2:** Comparison of the dampness syndrome scores as a function of the triglyceride level.

Hypertriglyceridemia	n	Dampness syndrome score	*F*	*P* value
None	5645	24.45 ± 16.22	7.46	<.001
Marginal elevation	1057	25.94 ± 16.99		
Mild to moderate elevation	887	26.89 ± 17.46		
Severe elevation	112	26.59 ± 17.67		

### 3.4. Comparison of dampness syndrome scores in different age groups

As shown in Table 3, the dampness syndrome scores in people between 20 and 40 years old increased with age, while the scores of dampness syndrome in people over 40 years old decreased with age, and the difference between age groups was statistically significant (*F* = 36.74, *P* ≤ 0.001).

**Table 3:** Comparison of dampness syndrome scores in different age groups.

Age (yr)	n	Dampness syndrome score	*F*	*P* value
20–29	676	24.93 ± 16.53	36.74	<.001
30–39	2056	27.18 ± 16.08		
40–49	2324	26.01 ± 16.79		
50–59	1785	24.15 ± 16.92		
60–69	660	18.94 ± 14.42		
70–79	200	17.49 ± 12.85		

### 3.5. Comparison of HTG among different demographic characteristics

As shown in Table 4, there were significant differences in the prevalence of HTG between different genders, marital status, and educational level (*P* < .001).

**Table 4:** Comparison of hypertriglyceridemia among different demographic characteristics.

Variable	n	Hypertriglyceridemia (%) (n)		*χ* ^2^	*P* value
		No	Yes		
Sex				415.26	<.001
Male	4834	65.4% (3161)	34.6% (1673)		
Female	2867	86.6% (2484)	18.6% (383)		
Marital status				99.96	<.001
Unmarried	976	86.1% (840)	13.9% (136)		
Divorced/widowed single	396	75.8% (300)	24.2% (96)		
Married	6120	71.4% (4367)	28.6% (1753)		
Remarry	209	66.0% (138)	34.0% (71)		
Educational level				51.31	<.001
Below high school	42	71.4% (30)	28.6% (12)		
High school or technical secondary school	196	65.3% (128)	34.7% (68)		
College or undergraduate	5388	71.4% (3847)	28.6% (1541)		
Master degree or above	2075	79.0% (1640)	21.0% (435)		

### 3.6. Comparison of TG levels as a function of the degree of dampness syndrome

The comparison of the TG levels as a function of the degree of dampness syndrome is shown in Table 5. With the exception of the extremely severe dampness syndrome group, the greater the degree of dampness syndrome, the higher the serum TG content; this relationship was statistically significant (*P* < .05). Gender stratification was then performed and the trend in the TG level in the male group was consistent with the overall trend, while the mean TG level in the females with severe dampness syndrome and above showed a downward trend (*P* < .05).

**Table 5:** Comparison of triglycerides levels of the sample as a function of the degree of dampness syndrome.

Degree of dampness syndrome	n (male/female)	Triglycerides (mmol/L)		
		Totality	Male	Female
None	3430 (2238/1192)	1.47 ± 1.27	1.67 ± 1.46	1.09 ± 0.64
Mild	2812 (1690/1122)	1.50 ± 1.33	1.73 ± 1.52	1.17 ± 0.87
Moderate	1184 (714/470)	1.61 ± 1.25	1.86 ± 1.41	1.22 ± 0.80
Severe	248 (174/74)	1.73 ± 1.31	1.99 ± 1.46	1.12 ± 0.43
Extremely severe	27 (18/9)	1.49 ± 1.22	1.81 ± 1.37	0.86 ± 0.42
*F*		4.21	3.56	3.23
*P* value		.002	.007	.012

### 3.7. Correlations between HTG, dampness syndrome, and chronic diseases

The correlations between HTG, dampness syndrome, and chronic diseases are shown in Table 6. Hypertriglyceridemia was significantly correlated with dampness syndrome, overweight/obesity, hypertension, diabetes, coronary atherosclerosis, carotid plaques, emphysema, thyroid nodules, and hyperuricemia (*P* < .05). According to gender stratification, the correlations between HTG, chronic diseases, and dampness syndrome was different in different genders.

**Table 6:** Correlations between hypertriglyceridemia, dampness syndrome, and chronic diseases.

Variable	Totality		Male		Female	
	*R*	*P* value	*R*	*P* value	*R*	*P* value
Dampness syndrome	0.038	.001	0.06	<.001	0.03	.113
Overweight/obesity	0.288	<.001	0.232	<.001	0.223	<.001
Hypertension	0.82	<.001	0.035	.016	0.153	<.001
Diabetes	0.081	<.001	0.045	.002	0.150	<.001
Coronary atherosclerosis	0.071	<.001	−0.020	.169	0.031	.094
Carotid plaques	0.073	<.001	0.026	.066	0.064	.001
Lacunar cerebral infarction	0.002	.883	0.047	.001	0.044	.018
Emphysema	0.045	<.001	−0.006	.664	0.026	.170
Thyroid nodules	0.030	.008	0.015	.296	0.015	.434
Hyperuricemia	0.260	<.001	0.040	.005	0.098	<.001

### 3.8. Correlations between HTG, demographic characteristics, and lifestyle factors

A description of the variables included in this analysis is provided in Table 7. The correlations between HTG, demographic characteristics, and lifestyle factors are shown in Table 8. Hypertriglyceridemia was significantly correlated with gender, age, marital status, education level, length of air conditioning in summer, smoking, frequency of drinking, frequency of night snack intake, structure of staple food consumption, frequency of fruit intake, frequency of milk/beans and milk/bean product intake, frequency of dessert or snack intake, daily time sitting, midday rest time, daily sleep duration and degree of TCM dampness syndrome(*P* < .05).

**Table 7:** Description of the demographic variables and lifestyle factors.

Characteristic	Assignment
Sex	Male = 1, female = 2
Age	Continuous variable
Marital status	Spinster = 0, divorce/widowed single = 1, married = 2, remarried = 3
Educational level	Below high school diploma = 0, high school or technical secondary school = 1, college or undergraduate = 2, master degree or above = 3
Working pressure	None = 0, little = 1, medium = 2, great = 3
Air conditioning time in summer	Never = 0, <4 h = 1, 4–8 h = 2, >8 h = 3
Ambient air quality	Fine = 0, light pollution = 1, moderate = 2, severe = 3
Smoking	Yes = 1, no = 0
Frequency of drinking	Abstain from alcohol = 0, <1 time/wk = 1, 1–2 times/wk = 2, >3 times/wk = 3, given up drinking = 4
Eat regularly	Occasionally = 0, most of the time = 1, everyday = 2
Frequency of breakfast	Never = 0, rarely (≤1–2 times/wk) = 1, sometimes (3–5 times/wk) = 2, almost every day (>5 times/wk) = 2
Frequency of night snack	Never = 0, occasionally = 1, frequently = 2
Staple food structure	Never = 0, predominantly roughage = 1, equilibrium = 2, predominantly fine grains = 3
Frequency of fruit intake	Never = 0, occasionally = 1, frequently = 3
Daily vegetable intake	<100 g = 0, 100–250 g = 1, 250–800 g = 2, >800 g = 3
Daily meat intake	<50 g = 0, 50–100 = 1, 100–200 g = 2, >200 g = 3
Beans and bean product intake	Rarely = 0, sometimes = 1, frequently = 2
Milk and milk product	Never = 0, rarely = 1, sometimes = 2, almost every day = 3
Dessert or snack intake	No = 0, occasionally = 1, frequently = 2
Exercise	No = 1, yes = 2
Daily sitting time	<4 h = 0, 4–8 h = 1, 8–12 h = 2, >12 h = 3
Work and rest time	Irregularity = 0, most often regularly = 1, regularly = 2
Frequency of midday rest time	No = 0, occasionally = 1, frequently = 2
Midday rest time duration	<15 min = 0, 15–45 min = 1, 46–90 min = 2,>90 min = 3
Time for bed	Before 21 = 0, 21 to 23 = 1, 23 to 1 = 2, after 1 = 3, not sure = 4
Daily sleep duration	<5 h = 0, 5.1–7 h = 1, 7.1–9 h = 2, >9 h = 3
Degree of dampness syndrome	None = 0, mild = 1, moderate = 2, severe = 3, extremely severe = 4

**Table 8:** Correlations between hypertriglyceridemia, demographic characteristics, and lifestyle factors.

Variable	*R*	*P* value
Sex	–0.232	<.001
Age	0.127	<.001
Marital status	0.107	<.001
Education level	–0.081	<.001
Working pressure	–0.021	.072
Air conditioning time in summer	–0.043	<.001
Ambient air quality	–0.014	.235
Smoking	0.169	<.001
Frequency of drinking	0.159	<.001
Eat regularly	0.003	.762
Frequency of breakfast	–0.011	.356
Frequency of night snacks	0.033	.004
Staple food structure	0.030	.008
Frequency of fruit intake	–0.036	.002
Daily vegetable intake	–0.018	.110
Daily meat intake	0.001	.947
Beans and bean product intake	–0.028	.015
Milk and milk product intake	–0.086	<.001
Dessert or snack intake	–0.069	<.001
Exercise	–0.001	.952
Daily sitting time	–0.033	.004
Work and rest time	–0.008	.507
Frequency of midday rest	–0.005	.658
Midday rest time duration	0.038	<.001
Time for bed	0.013	.246
Daily sleep duration	–0.030	.008
Degree of dampness syndrome	0.038	<.001

### 3.9. Binary logistic regression analysis of HTG and related indicators

**Figure 1.**
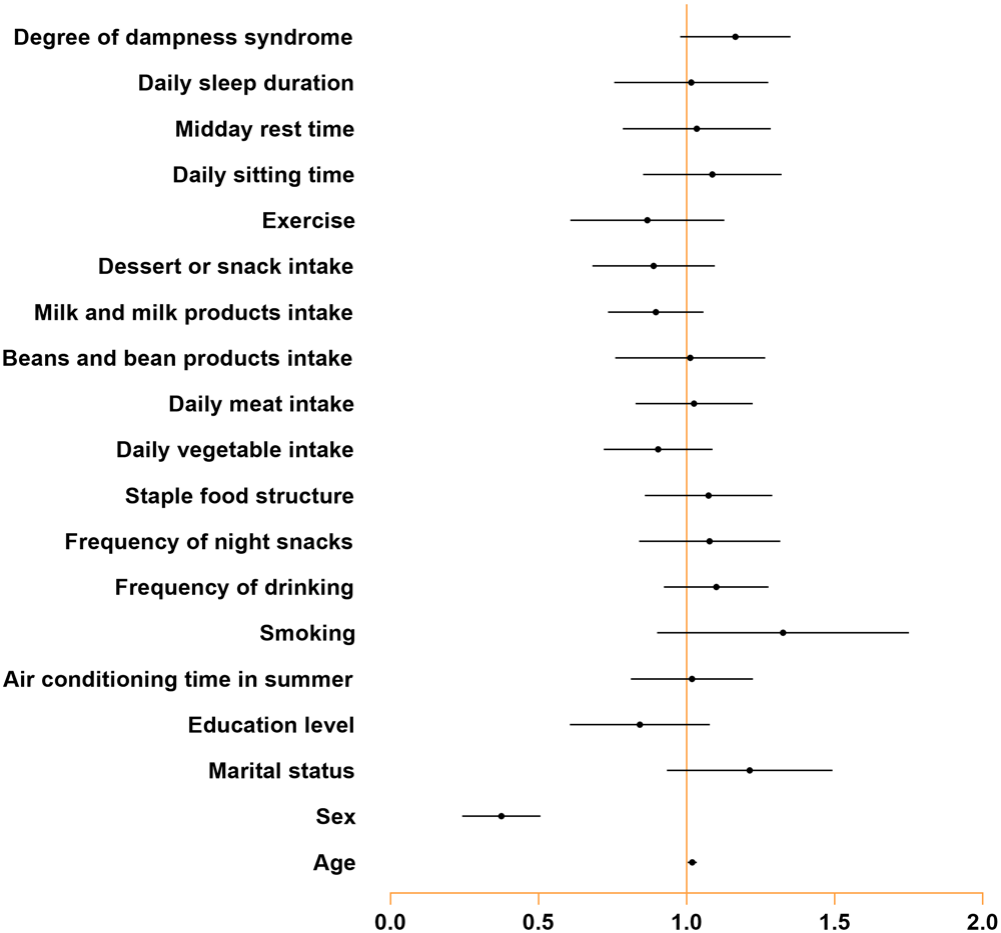
Binary logistic regression analysis of hypertriglyceridemia and related indicators.

The regression analysis of HTG and related indicators is shown in Table 9 and Figure 1. Binary logistic regression analysis was performed with HTG as the dependent variable and indicators such as dampness syndrome and lifestyle factors as the independent variables. The results revealed that age, sex, marriage, education level, frequency of smoking, frequency of drinking, frequency of fruit intake, daily intake of vegetables, frequency of milk and milk product intake, frequency of sweets intake, degree of TCM dampness syndrome and exercise were predictors of HTG (*P* < .05).

**Table 9:** Binary logistic regression analysis of hypertriglyceridemia and related indicators.

Variable	*β*	Wald	*P* value	Exp (B)	95% Confidence interval
Age	0.019	37.041	<.001	1.019	1.013–1.025
Sex	–0.989	186.085	<.001	0.372	0.323–0.429
Marital status	0.190	16.167	<.001	1.210	1.102–1.327
Education level	–0.176	9.9288	.002	0.839	0.749–0.939
Air conditioning time in summer	0.016	0.144	.704	1.016	0.936–1.102
Smoking	0.276	17.422	<.001	1.318	1.158–1.500
Frequency of drinking	0.094	8.112	.004	1.098	1.030–1.172
Frequency of night snacks	0.072	2.495	.114	1.075	0.983–1.175
Staple food structure	0.069	2.826	.093	1.072	0.989–1.162
Frequency of fruit intake	0.135	4.646	.031	1.145	1.012–1.294
Daily vegetable intake	–0.103	6.021	.014	0.902	0.831–0.979
Daily meat intake	0.023	0.339	.560	1.023	0.947–1.106
Beans and bean products intake	0.009	0.028	.868	1.009	0.912–1.116
Milk and milk products intake	–0.112	9.041	.003	0.894	0.832–0.962
Dessert or snack intake	–0.121	6.451	.011	0.886	0.807–0.973
Exercise	–0.147	5.670	.017	0.863	0.765–0.974
Daily sitting time	0.081	3.333	.068	1.084	0.994–1.182
Midday rest time	0.030	0.366	.545	1.031	0.935–1.136
Daily sleep duration	0.012	0.052	.820	1.012	0.913–1.122
Degree of dampness syndrome	0.151	21.116	<.001	1.163	1.090–1.240
Constant	–1.926	41.380	<.001	0.146	

## 4. Discussion

Dyslipidemia has become increasingly common among Chinese adults in recent years due to improvements in living conditions.^[15]^ HTG is a type of dyslipidemia. A study of adults in Wuhan, China, in 2019 reported that the prevalence of HTG was 13.9% (19.8% in men and 5.8% in women).^[16]^ In 2021, a health survey of 4598 adult residents in Shanxi Province found that the incidence of HTG reached 33.3%.^[17]^ In the current study, the prevalence of HTG was 26.7%. An elevated TG concentration has repeatedly been found to be a strong independent risk factor for cardiovascular disease^[18,19]^ and an important independent predictor of all-cause mortality for all races.^[20,21]^ Therefore, early identification of HTG is crucial for the prevention of cardiovascular disease and the reduction of all-cause mortality. TCM classifies substances such as TGs and BUA as “water and grain subtle,” and if they are not normalized, they will release harmful substances into the body.^[22]^ Epidemiological studies of the physical constitution have indicated that phlegm dampness and damp-heat constitution are closely related to metabolic disorders such as diabetes and hyperlipidemia.^[23,24]^ The study by Huang et al^[25]^ suggested that in phlegm-dampness, there is a reduction in the ability to clear blood lipids and regulate lipid metabolism, and thus, the phlegm-damp population is more likely to suffer from HTG.^[26]^ Previous studies paid more attention to the relationship between different constitutions and metabolic disorders in TCM, and rarely explored the relationship between dampness syndrome and HTG. In addition to focusing on the relationship between dampness syndrome and metabolism, this study further quantified the dampness syndrome score to explore the relationship between different degrees of dampness syndrome, HTG, and chronic diseases. The current study revealed that the average dampness syndrome score of the HTG group was higher than that of the normal group, and the dampness syndrome score increased with increasing degree of triglyceridemia (*P* < .05); although, the severely elevated group did not show an obvious increasing trend. Similarly, serum TG levels increased with increasing degree of dampness syndrome (*P* < .05). Moreover, regression analysis showed that the degree of TCM dampness syndrome was positively correlated with HTG and was an independent predictor of HTG, suggesting that the TCM dampness syndrome score could reflect the state of triglyceridemia to a certain extent. However, the TG content in the extremely severe dampness syndrome group did not show an increasing trend. This may be related to the small number of people in this group (only 27 cases). Further studies with larger sample sizes are needed to clarify the relationship between extremely severe dampness syndrome and TGs.

The current study also found that metabolic indicators such as BMI, SBP, DBP, FBG, and BUA were higher in the HTG group as compared to the normal group. Further analysis of the correlations between HTG and chronic diseases revealed that HTG was correlated with multiple chronic diseases such as overweight/obesity, hypertension, diabetes, and carotid plaques. This is consistent with previous reports. A study of Sri Lankan adults reported a positive correlation between serum TG levels and BMI.^[27]^ Peng et al^[28]^ reported that high TGs were significantly correlated with hyperuricemia. A prospective cohort study^[29]^ found that high TGs increased the incidence of hyperuricemia. Pan et al^[30]^ showed that up to 50% of overweight and obese people have HTG. Wyszyńska et al^[31]^ found that HTG is an independent risk factor for hypertension. Other studies have shown that in patients with type 2 diabetes, the main abnormality of lipid metabolism is a high TG level.^[32]^ In a recent study investigating, the association between dyslipidemia and diabetes, fasting TG levels were identified as an independent risk factor for the onset of diabetes (odds ratio: 2.33, 95% confidence interval: 1.61–3.39).^[33]^ Whether dampness syndrome is a risk factor for HTG and other diseases requires investigation in a future prospective study.

The results of the current study indicated that age, gender, marriage, education level, smoking, drinking, fruit consumption, vegetable consumption, milk and milk product consumption, sweets consumption, degree of TCM dampness syndrome, and exercise are predictors of HTG. This is consistent with previous research.^[34–37]^ For example, Spitler and Davies^[34]^ found that age-induced changes in human TG metabolism include an increased plasma TG level, decreased plasma TG clearance after meals, decreased lipolysis in adipose tissue, and increased ectopic fat deposition. In another study, an additional 100 g of ethanol intake per week increased serum TG by 0.041 mmol/L.^[38]^ A cross-sectional study of obese adults in Malaysia found that elevated TG levels were more prevalent in men, married people, and smokers.^[39]^ Vegetable intake has been found to be negatively correlated with increases in TG,^[40]^ and daily physical exercise has been found to effectively reduce the TG level.^[41]^ In short, a poor lifestyle can increase the TG level. Thus, HTG patients should maintain a healthy lifestyle to reduce their TG levels.

Increasing evidence from studies has shown that HTG has adverse effects on health, and rational and rapid medication intervention is necessary to delay the progression of related diseases. As an important part of Chinese traditional medicine, TCM has a long history and rich clinical experience, especially in chronic diseases, subhealth states, and individualized medical treatment. With the increasing demand for health and medical care, the research on the combination of clinical medicine and TCM has a broad application prospect and important social significance, which has a certain guiding significance for the clinical medication judgment of HTG. Countless clinical practices have proved that TCM does have efficacy,^[42,43]^ and exploring the relationship between TCM evidence and disease development, finding out the scientific nature of TCM dialectics and its biological basis can provide a basis for the foundation of modern medicine in TCM.

There are several strengths of this study that should be highlighted. First, to our knowledge, this is the largest population-based study of the association between HTG and dampness syndrome. This study found, for the first time, that the dampness syndrome score can reflect the state of HTG to a certain extent, and thus, quantifying “dampness” in TCM may offer an intuitive and noninvasive means to assess the TG level. However, this study also has some limitations that should be considered. First, this was a single-center cross-sectional study. The results indicated that HTG is correlated with dampness syndrome and several chronic diseases; however, the causal relationships between these variables could not be determined. Any causal relationships need to be confirmed in future prospective studies. In addition, this study excluded participants with missing data, which could potentially result in selection bias. Subsequently, we will continue to explore the relationship and changes between TCM dampness syndrome and HTG using a multicenter, prospective cohort study.

In summary, this study revealed a correlation between the TCM dampness syndrome score and HTG. Thus, the dampness syndrome score can be used as an auxiliary indicator for predicting HTG, which is noninvasive and rapid in clinical application. The results of this study also confirmed that HTG is associated with a variety of chronic diseases and lifestyle factors. We will follow up the enrolled HTG population at different time points to dig out the changes of HTG, chronic diseases, and dampness syndrome score over time, and look for the trends and patterns of changes, so as to be used for health guidance.

## Author contributions

**Methodology:** Hui Zhou, Xiangsheng Cai, Darong Wu, Hongli Zeng.

**Writing—original draft:** Hui Zhou.

**Writing—review & editing:** Hui Zhou.

**Resources:** Weizheng Zhang, Xiangsheng Cai, Shuo Yang.

**Data curation:** Aolin Liu.

**Investigation:** Aolin Liu, Xiaowen Zhou, Jianxiong Cai.

**Formal analysis:** Xiaowen Zhou, Jianxiong Cai.

**Conceptualization:** Hongli Zeng, Darong Wu.

**Supervision:** Hongli Zeng, Darong Wu.

**Validation:** Hongli Zeng, Darong Wu.

## Supplementary Material



## References

[1] Hernandez P Passi N Modarressi T. Clinical management of hypertriglyceridemia in the prevention of cardiovascular disease and pancreatitis. Curr Atheroscler Rep. 2021;23:72. 34515873 10.1007/s11883-021-00962-z PMC8436578

[2] Deng J Cai X Hao M. Calcium dobesilate (CaD) attenuates high glucose and high lipid-induced impairment of sarcoplasmic reticulum calcium handling in cardiomyocytes. Front Cardiovasc Med. 2021;8:637021. 33604360 10.3389/fcvm.2021.637021 PMC7884338

[3] Lee H Park J-B Hwang I-C. Association of four lipid components with mortality, myocardial infarction, and stroke in statin-naïve young adults: a nationwide cohort study. Eur J Prevent Cardiol. 2020;27:870–81. 10.1177/2047487319898571 32013600

[4] Miller M Stone NJ Ballantyne C. American Heart Association Clinical Lipidology, Thrombosis, and Prevention Committee of the Council on Nutrition, Physical Activity, and Metabolism. Triglycerides and cardiovascular disease: a scientific statement from the American Heart Association. Circulation. 2011;123:2292–333. 21502576 10.1161/CIR.0b013e3182160726

[5] Freiberg JJ Tybjaerg-Hansen A Jensen JS Nordestgaard BG. Nonfasting triglycerides and risk of ischemic stroke in the general population. JAMA. 2008;300:2142–52. 19001625 10.1001/jama.2008.621

[6] Langsted A Freiberg JJ Tybjaerg-Hansen A Schnohr P Jensen GB Nordestgaard BG. Nonfasting cholesterol and triglycerides and association with risk of myocardial infarction and total mortality: the Copenhagen City Heart Study with 31 years of follow-up. J Intern Med. 2011;270:65–75. 21198993 10.1111/j.1365-2796.2010.02333.x

[7] Ren S Liu YT Zhang J. Analysis on essence and therapeutic principle of “water, dampness, phlegm and drink. Chin J Basic Med Chin Med. 2021;27:13–6.

[8] Li J. in Great Dictionary of Chinese Medicine. Beijing: People’s Medical Publishing House, 2004:1773–74

[9] Liu Y Ren S Cao Q Zhang J. Recognition pattern of phlegm-dampness syndrome based on metabolomics. Liaoning J Tradit Chin Med. 2021;48:1–4.

[10] Wang W Yang Z. Discussion on pathogenesis and treatment of dampness-pathogenic layers. Chin Modern Distance Educ Tradit Chin Med. 2021;19:131–3.

[11] Zhang L Shi G Xiong J. Correlation analysis between dyslipidemia and traditional Chinese medicine physique type. Electronic J Pract Clin Nurs. 2019;4:154–6.

[12] Zhang B Lu Q. Study on the correlation between the level of blood lipids in hyperlipidemia and the identification and classification of traditional Chinese medicine physique. Yunnan J Tradit Chin Med. 2019;41:21–2.

[13] Committee, C. C. E. P. Hypertriglyceridemia and its cardiovascular risk management expert consensus. Chin J Cardiovasc Dis. 2017;45:108–15. 10.3760/cma.j.issn.0253-3758.2017.02.008 28260315

[14] Lu T Xie QW Cai JX. Construction and preliminary optimization of TCM dampness syndrome evaluation scale. Chin J TCM. 2021;62:1677–83.

[15] Song PK Man QQ Li H. Trends in lipids level and dyslipidemia among Chinese adults, 2002-2015. Biomed Environ Sci. 2019;32:559–70. 31488232 10.3967/bes2019.074

[16] Zhang M Wan Z-C Lv Y-M. Ten-year time-trend analysis of dyslipidemia among adults in Wuhan. Current Med Sci. 2022;42:1099–105. 10.1007/s11596-022-2630-4 PMC9573792 36245027

[17] Gao H Wang H Shan G. Prevalence of dyslipidemia and associated risk factors among adult residents of Shenmu City, China. PLoS One. 2021;16:e0250573. 33961634 10.1371/journal.pone.0250573 PMC8104371

[18] Forrester JS. Triglycerides: risk factor or fellow traveler. Curr Opin Cardiol. 2001;16:261–2644. 11574788 10.1097/00001573-200107000-00007

[19] Labreuche J Touboul PJ Amarenco P. Plasma triglyceride levels and risk of stroke and carotid atherosclerosis: a systematic review of the epidemiological studies. Atherosclerosis. 2009;203:331–45. 18954872 10.1016/j.atherosclerosis.2008.08.040

[20] Liu J Zeng F-F Liu Z-M Zhang C-X Ling W-H Chen Y-M. Effects of blood triglycerides on cardiovascular and all-cause mortality: a systematic review and meta-analysis of 61 prospective studies. Lipids Health Dis. 2013;12:159. 24164719 10.1186/1476-511X-12-159 PMC4231478

[21] Pikhart H Hubáček JA Peasey A Kubínová R Bobák M. Association between fasting plasma triglycerides, all-cause and cardiovascular mortality in Czech population. Results from the HAPIEE study. Physiol Res. 2015;64:S355–61. 26680668 10.33549/physiolres.933179

[22] Dai Y Bai H Zhang H Huang Z. Study on distribution of traditional Chinese medicine physique in overweight patients with hyperuric acid and hypertriglyceridemia. Bulletin Tradit Chin Med. 2018;17:51–3.

[23] Meng L Zhu YB Xu J. Analysis and study on TCM constitution characteristics of different obesity phenotypes. Chin J General Med. 2020;23:221–6.

[24] Wang Q. Series study on phlegm-dampness constitution and its application in prevention and control of metabolic chronic diseases. Tianjin Chin Med. 2020;31:4–8.

[25] Huang L Chen Y Sun C. Study on intestinal flora characteristics of health care workers with wet physique in Lingnan. Chin J Tradit Chin Med. 2021;36:7371–6.

[26] Wang Y. Study on the level of Hcy in patients with dyslipidemia and its correlation with traditional Chinese medicine physique. Guangzhou Univ Chin Me. 2020;4:1–46.

[27] Somasundaram N Ranathunga I Gunawardana K. High prevalence of overweight/obesity in Urban Sri Lanka: findings from the Colombo Urban Study. J Diabetes Res. 2019;2019:1–9. 10.1155/2019/2046428 PMC6893241 31886277

[28] Aslan V Akçay G. Investigation of endocrine disease frequency in dyslipidemia. Arch Basic Clin Res. 2023;5:185–93.

[29] Xu Y Dong H Zhang B Zhang J Ma Q Sun H. Association between dyslipidaemia and the risk of hyperuricaemia: a six-year longitudinal cohort study of elderly individuals in China. Ann Med. 2022;54:2402–10. 36053052 10.1080/07853890.2022.2118368 PMC9448407

[30] Pan XF Wang L Pan A. Epidemiology and determinants of obesity in China. Lancet Diabetes Endocrinol. 2021;9:373–92. 34022156 10.1016/S2213-8587(21)00045-0

[31] Wyszyńska J Łuszczki E Sobek G Mazur A Dereń K. Association and risk factors for hypertension and dyslipidemia in young adults from Poland. Int J Environ Res Public Health. 2023;20:982. 36673736 10.3390/ijerph20020982 PMC9858900

[32] Vergès B. Pathophysiology of diabetic dyslipidaemia: where are we. Diabetologia. 2015;58:886–99. 25725623 10.1007/s00125-015-3525-8 PMC4392164

[33] Charoensri S Turnsaket S Pongchaiyakul C. Hypertriglyceridemia as an independent predictor for ten-year incidence of diabetes in Thais. Vasc Health Risk Manag. 2021;17:519–25. 34511921 10.2147/VHRM.S326500 PMC8412820

[34] Spitler KM Davies BSJ. Aging and plasma triglyceride metabolism. J Lipid Res. 2020;61:1161–7. 32586846 10.1194/jlr.R120000922 PMC7397742

[35] Ye XF Miao CY Zhang W Ji LN Wang J-G; ATTEND Investigators. Alcohol intake and dyslipidemia in male patients with hypertension and diabetes enrolled in a China multicenter registry. J Clin Hypertens (Greenwich). 2023;25:183–90. 36660769 10.1111/jch.14638 PMC9903194

[36] Wang X Wang Y Xu Z. Trajectories of 24-hour physical activity distribution and relationship with dyslipidemia. Nutrients. 2023;15:328. 36678199 10.3390/nu15020328 PMC9860816

[37] Homer AR Fenemor SP Perry TL. Regular activity breaks combined with physical activity improve postprandial plasma triglyceride, nonesterified fatty acid, and insulin responses in healthy, normal weight adults: a randomized crossover trial. J Clin Lipidol. 2017;11:1268–79.e1. 28673802 10.1016/j.jacl.2017.06.007

[38] Würtz P Cook S Wang Q. Metabolic profiling of alcohol consumption in 9778 young adults. Int J Epidemiol. 2016;45:1493–506. 27494945 10.1093/ije/dyw175 PMC5100616

[39] Daud A Shahadan SZ Ibrahim M Isa MLM Deraman S. Prevalence and association between triglyceride level and lifestyle factors among Malay obese class I and II adults. Enfermería Clínica. 2018;28:310–5.

[40] Zhu R Fogelholm M Poppitt SD. Adherence to a plant-based diet and consumption of specific plant foods-associations with 3-year weight-loss maintenance and cardiometabolic risk factors: a secondary analysis of the PREVIEW intervention study. Nutrients. 2021;13:3916. 34836170 10.3390/nu13113916 PMC8618731

[41] Sanllorente A Soria-Florido MT Castañer O. A lifestyle intervention with an energy-restricted Mediterranean diet and physical activity enhances HDL function: a substudy of the PREDIMED-Plus randomized controlled trial. Am J Clin Nutr. 2021;114:1666–74. 34582548 10.1093/ajcn/nqab246

[42] Liu Z Liu Y Xu H. Effect of electroacupuncture on urinary leakage among women with stress urinary incontinence: a randomized clinical trial. JAMA. 2017;317:2493–501. 28655016 10.1001/jama.2017.7220 PMC5815072

[43] Huang K Zhang P Zhang Z. Traditional Chinese Medicine (TCM) in the treatment of COVID-19 and other viral infections: efficacies and mechanisms. Pharmacol Therap. 2021;225:107843. 33811957 10.1016/j.pharmthera.2021.107843 PMC8011334

