# Supplementary material for: Unraveling the link between hypertriglyceridemia, dampness syndrome, and chronic diseases: A comprehensive observational study
Source: Medicine (Baltimore). 2024 Aug 16;103(33):e39207. doi: 10.1097/MD.0000000000039207 (PMC11332762; doi:10.1097/MD.0000000000039207)
Supplement: Supplementary file 1 [file medi-103-e39207-s001.docx]

Unraveling the Link Between Hypertriglyceridemia, Dampness Syndrome, and Chronic Diseases: A Comprehensive Observational Study

**Table S1. Traditional Chinese medicine dampness syndrome evaluation scale**

| Number | Item | Choose the answer |
| --- | --- | --- |
| 1 | Body obesity | ① No ② light ③ medium ④ heavy ⑤ extremely heavy |
| 2 | Body trapped heavy | ① No ② light ③ medium ④ heavy ⑤ extremely heavy |
| 3 | Feel hot, but touch the skin is not hot | ① No ② light ③ medium ④ heavy ⑤ extremely heavy |
| 4 | Sticky sweat / Have trouble sweating | ① No ② light ③ medium ④ heavy ⑤ extremely heavy |
| 5 | Lethargy | ① No ② light ③ medium ④ heavy ⑤ extremely heavy |
| 6 | Feel drowsy/sleepy | ① No ② light ③ medium ④ heavy ⑤ extremely heavy |
| 7 | The head is as heavy as a bundle | ① No ② light ③ medium ④ heavy ⑤ extremely heavy |
| 8 | Greasy face or hair | ① No ② light ③ medium ④ heavy ⑤ extremely heavy |
| 9 | The face is dirty as dirt | ① No ② light ③ medium ④ heavy ⑤ extremely heavy |
| 10 | Excessive eye discharge | ① No ② light ③ medium ④ heavy ⑤ extremely heavy |
| 11 | Sticky mouth | ① No ② light ③ medium ④ heavy ⑤ extremely heavy |
| 12 | A bad smell from mouth | ① No ② light ③ medium ④ heavy ⑤ extremely heavy |
| 13 | Thirsty but unwilling to drink | ① No ② light ③ medium ④ heavy ⑤ extremely heavy |
| 14 | Excessive phlegm | ① No ② light ③ medium ④ heavy ⑤ extremely heavy |
| 15 | A feeling of fullness in the abdomen | ① No ② light ③ medium ④ heavy ⑤ extremely heavy |
| 16 | Loss of appetite | ① No ② light ③ medium ④ heavy ⑤ extremely heavy |
| 17 | Nausea or vomiting | ① No ② light ③ medium ④ heavy ⑤ extremely heavy |
| 18 | Waist trapped heavy | ① No ② light ③ medium ④ heavy ⑤ extremely heavy |
| 19 | Limb and joint pain | ① No ② light ③ medium ④ heavy ⑤ extremely heavy |
| 20 | The joints of the limbs are heavy | ① No ② light ③ medium ④ heavy ⑤ extremely heavy |
| 21 | Fecal unformed | ① No ② light ③ medium ④ heavy ⑤ extremely heavy |
| 22 | Frequent bowel movements | ① No ② light ③ medium ④ heavy ⑤ extremely heavy |
| 23 | Sticky stool | ① No ② light ③ medium ④ heavy ⑤ extremely heavy |
| 24 | Excessive leucorrhea/ moist scrotum | ① No ② light ③ medium ④ heavy ⑤ extremely heavy |
| 25 | Skin sores or sores | ① No ② light ③ medium ④ heavy ⑤ extremely heavy |
| 26 | Coated tongue thick | ① No ② light ③ medium ④ heavy ⑤ extremely heavy |
| 27 | Coated tongue greasy | ① No ② light ③ medium ④ heavy ⑤ extremely heavy |
| 28 | The tongue is slippery and watery | ① No ② light ③ medium ④ heavy ⑤ extremely heavy |
| 29 | white lines of foam on the tongue | ① No ② light ③ medium ④ heavy ⑤ extremely heavy |
| 30 | The tongue is fat and big | ① No ② light ③ medium ④ heavy ⑤ extremely heavy |
